# Lower mortality is observed among low birth weight young infants who have received home-based care by female community health volunteers in rural Nepal

**DOI:** 10.1186/s12884-017-1355-z

**Published:** 2017-07-11

**Authors:** Dinesh Neupane, Penny Dawson, Robin Houston, Liladhar Dhakal, Jaganath Sharma, KC Gargi, Christina Lagos, Vishnu Khanal, Shiva Raj Mishra, Per Kallestrup

**Affiliations:** 1 Center for Global Health, Department of Public Health, Aarhus University, Aarhus, Denmark; 2 JSI Research and Training Institute Inc, Kathmandu, Nepal; 3 Nepal Development Society, Chitwan, Nepal

**Keywords:** Low birth weight, Community health workers, Newborn, Nepal

## Abstract

**Background:**

There has been little success in attempts to reduce the proportion of births with low birth weight (LBW). However, deaths associated with LBW may be prevented with extra attention to warmth, feeding, and prevention or early treatment of infections. There are few studies on this in Nepal and in many other developing countries. This is a cohort study to evaluate the risk of deaths among LBW infants who received FCHV follow up visit for home-based care compared to those who did not receive in Rural Nepal.

**Methods:**

A cohort study design was used with data from the Morang Innovative Neonatal Intervention (MINI) program in Nepal. Relative Risk (RR) is calculated to compare LBW neonates who received FCHV follow up visit as compared to LBW neonates who did not receive visit.

**Results:**

Out of 51,853 newborn infants recorded in the MINI database, 2229 LBW neonates were included in the analysis. The proportion of deaths among those who received FCHV follow up visit and those who did not receive were 2% (95% CI: 1%; 2%) and 11% (95% CI: 6%; 18%) respectively(*P* < 0.001). The relative risk of death in LBW infants who received FCHV follow up visit was 84% less as compared to LBW infants who did not receive (RR = 0·16; 95% CI: 0·09, 0·29).

**Conclusion:**

The current study indicates that to save the lives of LBW young infants simple home-based measures implemented through trained health volunteers within the existing government health system may be effective when technically more sophisticated measures such as tertiary health centers, pediatricians, and expensive technology are limited.

## Background

Low Birth Weight (LBW) is defined by the World Health Organization (WHO) as weight at birth of less than 2500 g [1]. LBW which is caused by preterm birth or intrauterine growth retardation, constitutes only about 15% of children born, but accounts for 60–80% of neonatal deaths [2, 3]. The highest incidence is observed in South Asia, where an estimated 31% of neonates are born LBW, making up nearly half of the world’s LBW neonates [4]. The fourth Millennium Development Goal (MDG-4) commits the international community to reducing mortality in children less than five years of age by two-thirds between 1990 and 2015 [5]. The Nepal Demographic and Health Survey showed there was a minimal reduction in the proportion of LBW infants from 14% in 2006 to 12% in 2011 [6, 7]. As the majority of births occur at home [6], it is difficult to identify LBW births occurring in Nepal’s rural communities and to intervene and manage the consequences of LBW. Therefore, community based management of LBW is essential in order to achieve Sustainable Development Goal-3 related to child health.

There has been little success in the attempts to reduce the proportion of LBW births [8]. However, deaths from LBW can be prevented with extra attention to warmth, feeding, and prevention or early treatment of infections [9–11]. Over a period of eight years, a study in India showed that there was a 58% decline in case fatalities among LBW neonates who received these preventive measures [12]. An earlier study in Pune, India revealed that home-based management of neonates with LBW or preterm birth, babies with asphyxia, feeding problems or illness achieved a 25% reduction in neonatal mortality by providing advice on keeping the baby warm, exclusive breast-feeding and minimum handling of the baby [13].

Despite the importance of the adverse impact of LBW on child survival, there have been few prospective studies evaluating effectiveness of community based intervention through trained volunteers for LBW infants in Nepal and in other developing countries, largely because of the difficulties inherent in community based data collection and the higher proportion of births occurring at home [12] [13]. As a result, there is inadequate information about effectiveness of community based intervention preventing deaths among LBW infants from such settings. This has hindered the development of appropriate neonatal interventions in the developing countries, especially within existing government health programs. This study explores the impact of community based management of LBW infants on mortality within two months of age, by mobilizing Female Community Health Volunteers (FCHVs) from the Morang Innovative Neonatal Intervention (MINI) program, a prospective cohort study from Nepal. Infants 0 to 2 months were included in the study because Nepal’s IMCI guidelines at the beginning of the project (2005) only addressed illnesses in children from 2 months to 59 months [14].

FCHVs are local women with limited formal education, who are serving voluntarily within the government system. They receive basic training (18 days) and periodic refresher and program-specific training. Approximately 50,000 FCHVs work actively in all villages of Nepal and are supported by Facility-Based Community Health Workers [15]. Their coverage extends up to wards—the smallest subdivision in villages and municipalities of Nepal. In the MINI program, FCHVs were provided with five days training on the management of neonatal sepsis. Serving a ward ranging from 1000 to 3000 in population, each FCHV was in direct contact with expecting mothers. FCHVs resupplied iron and folic acid tablets to pregnant women, encouraged them to attend antenatal care at health facilities, and provided counseling on essential newborn care messages and danger signs for possible severe bacterial infections (PSBI) in young infants (less than two months). In addition, independent of MINI’s program model, FCHVs conducted monthly mothers’ group meetings in their communities to raise awareness on a broad range of health topics and to introduce any new programs.

## Methods

### Study setting

Nepal is a low-income country of 26.5 million inhabitants, 78% of which live on less than two dollars a day and 83% of which live in rural and often remote areas [16, 17]. Its diverse ecological regions and unstable political situation create challenges for systematizing healthcare delivery [18]. In Nepal, on average only 50% of pregnant mothers attend the recommended four antenatal care visits at health facilities, and only 45% of mothers attend postnatal care, and this failure to appear is a risk factor for negative pregnancy outcomes, including LBW newborns [6].

Morang Innovative Neonatal Intervention (MINI) program focused on community based management and treatment of illnesses of newborn children in the Morang district of southeastern Nepal from 2005 to 2009 [19]. Morang is the second most populous district of Nepal, after Kathmandu, yet 80% of the total population of 914,799 live in rural areas. The per capita yearly income for Morang was USD 297 and the overall literacy rate was reported as 56% (female 47% and male 67%) [20, 21]. Morang district has 65 Village Development Committees (VDCs), two hospitals, seven primary health care centers, ten health posts, and 49 sub-health posts. There were 585 FCHVs in the District at the time of the study.

### Study design

MINI was a prospective intervention study aimed at managing newborn’s illnesses at household and community level. MINI began in 21 randomly selected VDCs in Morang, but due to initial successes and demand from local government from the VDC level and the District Public Health Office (DPHO) level, it was rapidly scaled-up to the entire Morang district covering all 65 VDCs. This is a cohort study utilizing the data from the MINI program to evaluate the risk of deaths in LBW infants who received follow up FCHV visit or not.

FCHVs were trained to measure birth weight on their initial visit and to do follow-up visits to advise on care of LBW babies. The choice to do a follow-up visit was voluntary and they did not receive any incentive for doing such follow-up visits. During the first visit, FCHVs recorded date of birth, birth place, birth condition, weight and assessed for any infection, as well as providing counseling on essential newborn care. For LBW babies, they conducted four additional follow-up visits on a weekly basis. On each follow up visit, they reassessed the young infant for signs of Possible Severe Bacterial Infection (PSBI) [22, 23], advising mothers to seek care immediately if any of these signs appeared, and counseling the mother on exclusive and frequent breastfeeding and skin-to-skin contact. FCHVs used topical antibiotics for Local Bacterial Infection (LBI- eye and skin infection) and initiated treatment with oral Cotrimoxazole-P and referred to facility-based health workers for Gentamicin injections for PSBI [14, 24]. FCHVs were already familiar with using Cotrimoxazole-P for treatment of pneumonia in older children and this antibiotic was successfully used in earlier study [24]. The infants referred for PSBI continued to get weekly follow up visits.

### Data collection

During training of FCHVs in MINI, they were provided with Salter scales and trained to measure the weight of newborns. Given the varying levels of literacy among FCHVs (38% of FCHVs in Nepal are illiterate) [15], MINI modified the Salter scale by coding weight ranges with colors to simplify measurements. The red, yellow and green portions of the scale signified Very Low Birth Weight (VLBW), LBW and normal birth weight respectively. Newborns weighing <2000 g were categorized VLBW (red color), those weighing between 2000 g – 2500 g were categorized LBW (yellow), and those weighing >2500 g were considered normal weight (green). If FCHVs circled the VLBW image in the birth weight section, they referred the infant and family to the nearest health facility. If the family did not comply with health facility referral, FCHVs visited the home, as they would for a LBW case, and marked the visit number on the recording form. Families with LBW babies were counseled further on newborn care. The FCHV then conducted four additional home visits (weekly for one month) for the LBW neonates.

Regardless of FCHVs follow up visits, the FCHV conducted a two-month (in 60 or more days) visit to determine the infant’s survival status at 60th day. MINI’s trained field supervisors collected and consolidated data from FCHVs on a regular basis. Standard forms and formats were developed and used for this purpose.

### Statistical analysis

Any infants with birth weight measured three days after birth, weight not-taken or survival status not known at the two-month were excluded from the analysis in order to maintain data consistency. Similarly, for comparing infants who received no follow visit and those who received at least one follow up visit by the FCHV for LBW infants, neonatal deaths within one week of birth were excluded as these population were not able to receive intervention from FCHVs. Furthermore, those infant (*n* = 97), who were referred and taken to health facility received advanced care from referral hospital, were excluded from analysis as including them would over-estimate the effect of FCHVs visit. Ethnicity classification was done based on the Health Management Information System (HMIS) of the Government of Nepal, which categorizes ethnicity in six categories namely, (i) Dalits, (ii) Disadvantaged Janajatis, (iii) Disadvantaged Non-Dalit Terai Caste Groups, (iv) Religious Minorities, (v) Relatively Advantaged Janajatis, and (vi) Upper Caste Groups [25]. Chi-square test was used to identify statistical difference in mortality outcomes between FCHV-visited and not-visited LBW neonates. Relative risk was calculated after adjusting for birth place to measure the relative risk reduction of death among LBW neonates who received an FCHV follow up visit compared to those who did not. *P*-values <0·05 were considered statistically significant.

### Ethical considerations

Informed oral consent of the infant’s legal guardian was obtained. The Ministry of Health and Population, Nepal approved the intervention. Ethical approval to conduct this study was obtained from the Western Institutional Review Board (WIRB) (WIRB Pro No. 20031870).

## Results

### Characteristic of samples

**Fig. 1:**
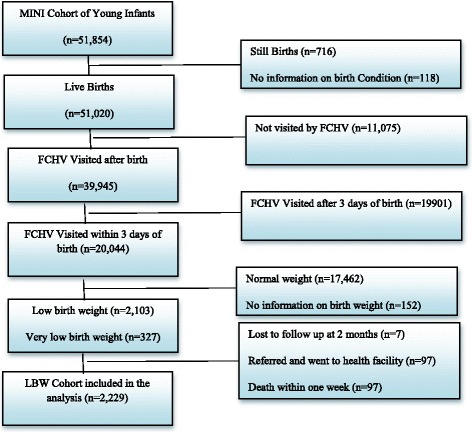
The exclusion and inclusion criteria of the study

The exclusion and inclusion criteria for this study are presented in Fig. 1. Out of 51,854 young infants recorded in the MINI database, 51,020 were live births, 716 were stillbirths and 118 did not have any information on birth condition. Among the live births, 11,075 did not have any information on birth weight and for 19,901 weights were measured after three days. Among 20,044 live births visited by FCHVs within three days, 17,462 had normal weight, 2103 had low birth weight, 327 had very low birth weight and 152 did not have any information on birth weight. From the low birth weight cohort, including very low birth weight, seven were lost to follow up, 97 were taken to a health facility after the referral by FCHV and 97 died within a week. The remaining 2229 births were included in the analysis.

The detailed characteristics of the young infants who were included in the analyses are presented in Table 1. Among 2229 low birth weight young infants, 53% were female. Ethnically, the majority of the infants were Disadvantaged Janajati (40%) followed by Disadvantaged Dalit (21%). There were 49 (2%) deaths between eight days and two months of age. The majority of births occurred at home (78%). Two hundred and eighty-six young infants had PSBI, among them 177 were identified by FCHVs and initiated treatment with oral antibiotic and referred for injectable antibiotics. The remaining 109 PSBIs were diagnosed by the local health workers from the health facilities. Likewise, 464 young infants had LBI and were treated by FCHVs using topical antibiotics. Among the 2229 LBW infants, 121 (5·4%) received no follow-up visits. No statistically significant differences between those with and without follow-up visits were observed by sex (*P* = 0·09), illness (*P* = 0·37), first place of treatment (*P* = 0·59) and ethnicity (*P* = 0·48). However, these two groups were s significantly different by birth-place (*p* = 0.03) (Table 1).

**Table 1:** Baseline characteristics of low birth weight infants

Variables	Total	Follow up visit	No Follow up visit	*P*-value
	(*n* = 2229)	(*n* = 2108)	(*n* = 121)	
Sex				
Male	1040 (47%)	975 (46%)	65 (54%)	0.095
Female	1189 (53%)	1133 (54%)	56 (46%)	
Ethnicity				
Upper Caste	315 (14%)	298 (14%)	17 (14%)	
Disadvantaged Janajati	959 (40%)	924 (44%)	52 (43%)	
Disadvantaged Non-Dalit Terai	178 (8%)	167 (8%)	11 (9%)	
Dalit	468 (21%)	449 (21%)	19 (16%)	
Religious Minority (Muslim)	178 (8%)	165 (8%)	13 (11%)	0.481
Others	114 (5%)	105 (5%)	9 (7%)	
Illness				
Yes	784 (34%)	746 (35%)	38 (31%)	0.372
No	1445 (66%)	1362 (65%)	83 (68%)	
Birth Place				
Home	1742 (71%)	1657 (79%)	85 (70%)	
Health Facility	487 (29%)	451 (21%)	36 (30%)	0.03
First place of treatment (*n* = 784)	784	746	38	
Home	194 (25%)	187 (25%)	7 (18%)	
Government Health Facility	136 (17%)	128 (17%)	8 (21%)	
Private Health Facility	49 (6%)	45 (6%)	14 (11%)	
Traditional Healers	14 (1%)	13 (2%)	1 (3%)	
FCHV	391 (50%)	373 (50%)	18 (47%)	0.590^a^

^a^Fischer exact test

**Fig. 2:**
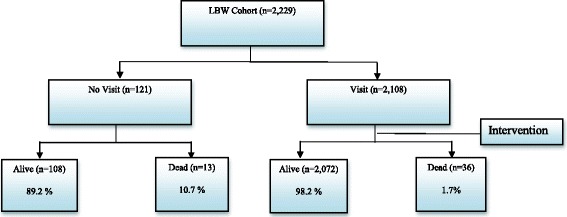
Mortality status at two months in LBW infants

Mortality status at 60th day in LBW infants are shown in Fig. 2. The birth place adjusted proportion of deaths among those who received FCHV follow up visit and those who did not receive were 2% (95% CI: 1%; 2%) and 11% (95% CI: 6%; 18%) respectively (*P* < 0.001). Further disaggregation of data showed the highest difference was observed for Dalits (27% vs 1·1%) and Disadvantaged Non-Dalit Terai Caste ethnic groups (27·0% vs 2·0%). The relative risk of death in LBW infants who received FCHV follow up visit was 84% less as compared to LBW infants who did not receive (RR = 0·16; 95% CI: 0·09, 0·29).

## Discussion

The results demonstrated that community based management of low birth weight infants within an existing government health system of Nepal is effective in reducing risk of deaths in LBW infants. The study showed that the LBW newborns who received at least one follow up visit by FCHVs had lower risk of mortality at two months of age compared to those who received no follow up visits. It appears that MINI’s home-based care by FCHVs which included a combination of weighing neonates within three days of birth, assessing neonates for danger signs, treating and referring sick neonates, encouraging exclusive breastfeeding, promoting skin-to-skin contact, counseling on recognition of danger signs and prompt care seeking in the event of PSBI or LBI reduces mortality among LBW infants. It has been demonstrated in other studies that promoting immediate drying and wrapping of the baby, hygienic cord tying and cutting, skin-to-skin contact with the mother, and initiation of breastfeeding within the first hour of birth are critical for improving the survival of newborn infants, especially those who have a low birth weight [26].

Concerns raised about the home-based or community based management of newborns often point to the risks involved in treating critical infants in settings so far removed from tertiary health facilities. While the ideal scenario would allow mothers to easily access these types of facilities, the reality in rural and low-resource settings is that the distance, cost and access to such services is out of reach for most families. Given these circumstances, services such as those provided by FCHVs in Nepal are critical for bringing essential newborn care closer to the home where more than 60% of deliveries occur [6]. Findings from our analysis provide further indications that it may be possible to save newborn lives in resource-poor settings by implementing home-based newborn care delivered by community health workers, and by promoting perinatal care at grass root level health facilities [27]. Another important finding of our study is that even the most deprived ethnic groups such as Dalit and non-Dalit Terai Caste Groups had a much lower mortality if they had received at least one follow visit. This is an important finding also from a program-implementation perspective because Dalits and Terai Madhesi groups continue to experience higher relative levels of neonatal mortality [28].

The integration of essential newborn care practices such as newborn weighing and follow-up visits are an important component for reducing young infant mortality. In the implementation process, it was observed that in addition to early antenatal contact through existing government health programs, weighing the newborn was an important entry point for identifying danger signs in infants. Mothers notified birth to FCHVs when they were aware that part of the first postnatal care visit would involve weighing of the newborn. By identifying births early and providing extra visits for LBWs, there was also an increased opportunity to identify PSBI cases in the home. This identification is critical for intervening through early treatment since neonatal sepsis often progresses rapidly and has a very high mortality [29].

There are several limitations to the current study. As the program was implemented in all VDCs of Morang district with no control group, the internal comparison between receiving at least one visit and no visit may not be representative to the population of infants who could benefit from this study. The visit by an FCHV may not be the sole factor contributing to a reduction in mortality in neonates. We do not have documented information on why the non-exposed did not get follow up-visits. However, the opportunities for receiving care by FCHVs for those who received care and those who did not receive care was comparable because FCHVs were distributed homogeneously in their community. The date of the follow-up visits is not available as the FCHVs just circled the number of the visit. Furthermore, limited data collection restricted conclusions on whether or not visits had a direct impact on increased weight at the end of the two-month period since young infants were not reweighed after they were identified as LBWs.

Because of the observational nature of this study, data on reasons for no follow up visits are unavailable. However, we can speculate that no follow-up visit may be related to geographical obstacles and long distances which prevented FCHVs from frequently visiting clients, cultural elements such as postpartum practices that lead a new mother to move from her in-laws’ home to her paternal home, and/or mothers’ education and awareness of LBW as a neonatal health issue [30].

Despite uncertainties related to the characteristics of both those receiving care and providing care for LBWs, MINI showed that LBW infants who received an FCHV visit experienced a significantly reduced risk of death compared to those who did not. The current study suggests that, to save the life of LBW infants simple home-based measures implemented through trained health volunteers within the existing government health system is effective when technically more sophisticated measures such as tertiary health centers, pediatricians, and expensive technology are scarce.

## Conclusions

Findings from the community based management and treatment of illness of newborns showed that there was lower risk of death among LBW infants who received home-based care from FCHVs. This indicates that home-based newborn care by trained health volunteers might have a significant impact in reducing young infant mortality in resource poor settings such as Nepal.

## Abbreviations

DPHO: District Public Health Office
FCHV: female community health volunteers
LBW: low birth weight
MDG: millennium development goal
MINI: Morang innovative neonatal intervention
PSBI: possible severe bacterial infections
RR: relative risk
USD: United States Dollar
VDC: Village Development Committee
WIRB: Western Institutional Review Board
